# Inhibition of COX-2, mPGES-1 and CYP4A by isoliquiritigenin blocks the angiogenic Akt signaling in glioma through ceRNA effect of miR-194-5p and lncRNA NEAT1

**DOI:** 10.1186/s13046-019-1361-2

**Published:** 2019-08-22

**Authors:** Chenlong Wang, Yaxin Chen, Yang Wang, Xiaoxiao Liu, Yanzhuo Liu, Ying Li, Honglei Chen, Chengpeng Fan, Dongfang Wu, Jing Yang

**Affiliations:** 1 Department of Pharmacy, Zhongnan Hospital of Wuhan University, Donghu Road 169, Wuhan, 430071 China; 2 Department of Pharmacology, School of Basic Medical Sciences, Wuhan University, Donghu Road 185, Wuhan, 430071 China; 3 Department of Pathology and Pathophysiology, School of Basic Medical Sciences, Wuhan University, Wuhan, China; 4 Department of Biochemistry and Molecular Biology, School of Basic Medical Sciences, Wuhan University, Wuhan, China

**Keywords:** Angiogenesis, Flavonoids, Arachidonic acid, Metabolism, ceRNA, Akt

## Abstract

**Background:**

Arachidonic acid (AA) metabolic enzymes including cyclooxygenase-2 (COX-2), microsomal prostaglandin E synthase-1 (mPGES-1) and cytochrome P450 (CYP) 4A11 play important roles in glioma angiogenesis. Thus, there is an urgent need to identify the underlying mechanisms and develop strategies to overcome them.

**Methods:**

A homology model of human CYP4A11 was constructed using SYBYL-X 2.0. Structure-based virtual screening against COX-2, mPGES-1 and CYP4A11was performed using the Surflex-Dock of the SYBYL suite. The candidates were further evaluated their antiangiogenic activities in a zebrafish embryo and rabbit corneal angiogenesis model. Laser doppler analysis was used to measure tumor perfusion. The expression of CD31 and α-SMA was measured by immunofluorescence. Western blot was used to measure the expression of HIF-1, Akt and p-Akt. The gene expression of FGF-2, G-CSF, PDGF, TGF-β, Tie-2, VEGF, lncRNA NEAT1 and miR-194-5p were determined using qPCR. The production of FGF-2, TGF-β and VEGF were analyzed using ELISA. Bioinformatic analysis and luciferase reporter assays confirmed the interaction between lncRNA NEAT1 and miR-194-5p.

**Results:**

The nearly 36,043 compounds from the Traditional Chinese Medicine (TCM) database were screened against COX-2, mPGES-1 and CYP4A11 3D models, and the 17 top flavonoids were identified. In zebrafish screening, isoliquiritigenin (ISL) exhibited the most potent antiangiogenic activities with the EC_50_ values of 5.9 μM. Conversely, the antiangiogenic effects of ISL in the zebrafish and rabbit corneal models were partly reversed by 20-hydroxyeicosatetraenoic acid (20-HETE) or prostaglandin E2 (PGE_2_). ISL normalized glioma vasculature and improved the efficacy of temozolomide therapy in the rat C6 glioma model. Inhibition of COX-2, mPGES-1 and CYP4A by ISL decreased FGF-2, TGF-β and VEGF production in the C6 and U87 glioma cells with p-Akt downregulation, which was reversed by Akt overexpression. Furthermore, ISL downregulated lncRNA NEAT1 but upregulated miR-194-5p in the U87 glioma cell. Importantly, lncRNA NEAT1 overexpression reversed ISL-mediated increase in miR-194-5p expression, and thereby attenuated FGF-2, TGF-β and VEGF production.

**Conclusions:**

Reprogramming COX-2, mPGES-1 and CYP4A mediated-AA metabolism in glioma by flavonoid ISL inhibits the angiogenic Akt- FGF-2/TGF-β/VEGF signaling through ceRNA effect of miR-194-5p and lncRNA NEAT1, and may serve as a novel therapeutic strategy for human glioma.

**Electronic supplementary material:**

The online version of this article (10.1186/s13046-019-1361-2) contains supplementary material, which is available to authorized users.

## Background

Angiogenesis, the sprouting of new blood vessels from an existing vascular network, is integral to the pathology of conditions such as cancer [1]. Tumor vasculature is characterized by dilation, tortuosity, leakiness and loss of hierarchical architecture, which contributes to tumor hypoxia and progression [2]. Multiple angiogenesis inhibitors have been therapeutically validated in both preclinical and clinical settings, but concomitantly elicit tumor adaptation and progression to stages of greater malignancy, with heightened invasiveness and increased metastasis [3]. It appears that clinical application of antiangiogenic therapy is more complex than originally thought [4]. However, vascular normalization, occuring in the context of antiangiogenic treatment, offers chances to normalize tumor microenvironment and ultimately improves the therapeutic efficacy of anticancer drugs, which contributes to a better outcome [5].

Metabolites from arachidonic acid (AA), such as prostaglandin E_2_ (PGE_2_) and 20-hydroxyeicosatetraenoic acid (20-HETE), are thought to be important mediators in tumor angiogenesis [6, 7]. Cyclooxygenase (COX)-2 inhibition potentiates antiangiogenic cancer therapy in preclinical models [6]. Knockdown of microsomal PGE synthase (mPGES)-1 inhibits angiogenesis in the B16 melanoma [8]. Our previous study showed that cytochrome P450 (CYP) 4A11-derived 20-HETE promotes lung cancer angiogenesis by the upregulation of VEGF [9], whereas inhibition of 20-HETE synthesis by N-hydroxy-N-(4-butyl-2 methylphenyl)-formamidine (HET0016) blocks the angiogenic responses to EGF, VEGF and FGF-2 in human glioma cell U251 and lung cancer cell A549 [9, 10]. Unfortunately, when COX-2 or CYP4A is blocked, AA metabolism would be switched to the other pathway, which will decrease the efficacy and exacerbate adverse effects [11]. Thus, the combination inhibition of COX-2, mPGES-1 and CYP4A could represent a promising therapeutic strategy for tumor angiogenesis.

Epidemiological study showed an inverse relationship between consumption of flavonoids and cancer risk [12]. A flavanes-type flavonoid Epigallocatechin-3-gallate suppresses angiogenesis in a C6 glioma model, and induces vascular normalization in non-small cell lung cancer [13]. A chalcone-type flavonoid isoliquiritigenin (ISL) inhibits breast cancer neoangiogenesis via VEGF signaling [14]. Flavonoid silibinin inhibits colorectal cancer growth and angiogenesis through the downregulation of COX-2 [15]. Our previous study showed that flavonoid FLA-16 prolongs survival and normalizes tumor vasculature through the downregulation of CYP4A in glioma [16]. Calycosin inhibits the in vitro and in vivo growth of breast cancer cells through lncRNA WDR7–7 and miR-375 [17]. Despite these encouraging results, multi-target inhibitors from flavonoids that inhibit both COX-2/mPGES-1 and CYP4A pathway through ceRNA effect will be beneficial in the prevention and treatment of tumor angiogenesis.

In this study, 36,043 compounds from the Traditional Chinese Medicine (TCM) database against the crystal structure of human COX-2 (PDB id: 5IKT) and human mPGES-1 (PDB id: 5BQG) and the homology model of human CYP4A11 were first screened, respectively. The docking results were then evidenced by enzyme inhibition assays, zebrafish and rabbit cornea angiogenesis models. Finally, the vascular normalization effects of the candidates were investigated in a rat C6 glioma model. In particular, we investigated whether the triple inhibitor of COX-2, mPGES-1 and CYP4A blocks tumor angiogenesis through ceRNA effect. Our results could lead to a novel therapeutic strategy for human glioma.

## Methods

### Reagents

The hit compounds were purchased from Ambinter (Orléans, France), Analyticon Discovery (Postdam, Germany) and Sigma-Aldrich (St. Louis, MO), and their purity was ≥95% as determined by HPLC analysis. WIT003 (an analog of 20-HETE) was synthesized and provided by Dr. John R. Falck. 20-HETE was purchased from Cayman Chemicals (Ann Arbor, MI). The antibodies against rat CD31, α-smooth muscle actin (SMA) and hypoxia-inducible factor (HIF)-1α were purchased from Santa Cruz Biotechnology, Inc. (Texas, USA). IgG/horseradish peroxidase was purchased from Kirkegaard & Perry Laboratories, Inc. (Gaithersburg, MD). The antibodies against VEGF and β-actin were purchased from Abcam, Inc. (Cambridge, MA). For in vivo experiment, liposomal ISL was prepared by mixing ISL, DMPC and cholesterol at a mole ratio of 1:5:5 according to a modification of Lim et al. (2000). The final products were stored at − 20 °C and warmed up to room temperature just before use.

### Homology modeling

The homology model of human CYP4A11 was automatically generated by the SWISS-MODEL program using the crystal structure of human CYP4B1 (PDB id: 5T6Q) as a template [18]. The heme was manually merged into the protein to occupy the same position as the heme of the template protein (CYP4B1) using the Coot 0.8.2 program [19]. Subsequently, a 6 ns dynamic simulation was performed using GROMACS 4.5.4 software (http://www.gromacs.org) [20]. The quality of the final model was validated by two programs, Procheck and Verify_3D, both of which belong to the structure analysis-validation online server sponsored by the UCLA-DOE Institute for Genomics and Proteomics [21].

### Virtual screening

The crystal structure of human COX-2 (PDB id: 5IKT) and human mPGES-1 (PDB id: 3DWW) were retrieved from the protein data bank (PDB). The homology model of human CYP4A11 was automatically generated by the SWISS-MODEL program using the crystal structure of *Oryctolagus cuniculus* CYP4B1 (PDB id: 5T6Q) as a template. The molecular docking study was performed using the Surflex-Dock of SYBYL-X 2.0 (Tripos, St. Louis, MO). The SYBYL software was used to assign the standard AMBER atomic partial charges on the COX-2, mPGES-1 and CYP4A11 protein and the Gasteiger-Hückel atomic partial charges on the ligand candidates to be docked. After the preparation, the docking was performed using the default settings, and the figures were generated using PyMol (http://www.pymol.org).

### Cell cultures

The C6 glioma cell line was purchased from the American Type Culture Collection (ATCC, Manassas, VA). The cells were grown in Dulbecco′s modified Eagle medium containing 10% fetal bovine serum in a humidified atmosphere of 5% CO_2_–95% air at 37 °C.

### Cellular thermal shift assay (CETSA)

CETSA was conducted using cell lysates as previously described [22]. For the temperature-dependent thermal shift assay, 50 μL of lysates (3 mg/mL) from U87 cells were incubated with 20 μM of ISL at each temperature point from 36 to 80 °C for 4 min. The supernatant and pellet were separated from the above samples by centrifugation at 20,000 g for 10 mins. 12 μL of the supernatant was mixed with 3 μL of 5 × loading buffer and then separated on a 10% SDS-PAGE for immunoblotting analysis of COX-2, mPGES-1 or CYP4A11. For the dose-dependent thermal shift assay, 50 μL of lysates (3 mg/mL) were incubated with various concentrations of ISL (between 0.001 to 1000 μM) at 52 °C for 4 min. Supernatants were isolated by centrifugation and subjected to immunoblotting analysis of COX-2, mPGES-1 or CYP4A11 as described above.

### Zebrafish embryo angiogenesis assay

Zebrafish embryo angiogenesis model was performed as previously described [23]. Zebrafish embryos in 96-well microplates were treated with the indicated concentrations of compounds (10 μM) alone, the compounds (10 μM) plus 20-HETE or PGE_2_, and vehicle for 48 h, and fixed in 4% paraformaldehyde for 2 h at room temperature. The embryos were stained for 10 min with 4.5 μl of 75 mg/ml nitroblue tetrazolium and 3.5 μl of 50 mg/ml 5-bromo-4-chloro-3-indolyl phosphate. All the blood vessels in the embryos were photographed with a stereomicroscope for analysis by image pro plus 6.0 (Media Cybernetics, Inc. WY, USA).

### Rabbit corneal neovascularization assay

Rabbit corneal neovascularization model was performed as previously described [24]. A 7–0 black silk was used to pass the rabbit corneal stroma 1 mm from the horizontal to the limbus with a length of 3 mm. One week after suturing, the suture was removed, and the compound ISL (2.0 mg/ml), ISL (2.0 mg/ml) plus 20-HETE or PGE_2_, and vehicle were performed twice a day for a week in eye drops. Photographs were taken with a stereomicroscope at day 0, 3 and 7 after treatment, and the vessel areas were quantified using image pro plus 6.0.

### Tumor models and treatment regimes

Wistar rats (male, 6–8 weeks old) were provided by the Experimental Animal Center of Wuhan University, housed on a 12-h light/12-h dark cycle in a pathogen-free environment, and allowed ad libitum access to food and water. All animal studies were approved by the Animal Research Committee of Wuhan University, and maintained in accordance with the guidelines by the Association for Assessment and Accreditation of Laboratory Animal Care International. For the subcutaneous tumor model, 200 μl cell suspension containing 5 × 10^6^ rat C6 glioma cells was injected subcutaneously into the right flank. When tumors reached a size of about 100 mm^3^, ISL was administered intraperitoneally at the dose of 10, 20 mg/kg once daily. The rats were euthanized 24 h after completion of treatment. Tumors were removed and weighed. The in vivo doses were selected on the basis of preliminary experiments which demonstrated the absence of organ toxicity induced by the treatments.

To evaluate the effects of ISL on chemotherapy, temozolomide (TMZ) was injected intraperitoneally either as a single dose of 20 mg/kg after treatment with ISL (20 mg/kg) for 7 days to determine the concentration of TMZ in tumor tissues or at a dose of 20 mg/kg once daily for 7 consecutive days to evaluate the tumor growth and TMZ concentration.

### Laser Doppler analysis of tumor perfusion

The tumor perfusion was measured by laser Doppler analysis as previously described [16, 25]. Briefly, the C6-bearing rats as described above were anesthetized with 0.6% pentobarbital and placed on a heating pad (37 °C). The cutaneous envelope over each tumor was carefully excised, protecting the vascular network decorating the tumor mass. Tumor perfusion in the rats treated with ISL (10, 20 mg/kg) or vehicle at day 0, 2, 4, 6 and 8 was blindly measured using a laser Doppler analyzer (LDPI; Moor Instruments). The tumor perfusion in arbitrary perfusion units was monitored graphically.

### Quantification of 20-HETE

20-HETE was analyzed by liquid chromatographic-tandem mass spectrometry (LC-MS/MS) as previously described [16]. Each experiment was repeated for 3 times, and all samples were analyzed in triplicate.

### Enzyme inhibition assay

The rat renal microsomes were prepared as previously described [26]. Arachidonic acid was incubated with the microsomal protein in the presence or absence of the candidates for 2 min, and 20-HETE was analyzed as described above. The IC_50_ of the candidates for CYP4A-dependent fatty acids hydroxylation was determined using GraphPad Prism (GraphPad Software Inc., San Diego, CA).

### Statistical analysis

All values are expressed as mean ± S.E.M., and statistical analyses were performed using a one-way ANOVA followed by the Student-Newman-Keul’s test. Values were compared using multiple comparisons, where *P* values of 0.05 or less were considered significant.

## Results

### Structure-based virtual screening against COX-2, mPGES-1 and CYP4A11 and zebrafish screening

**Fig. 1:**
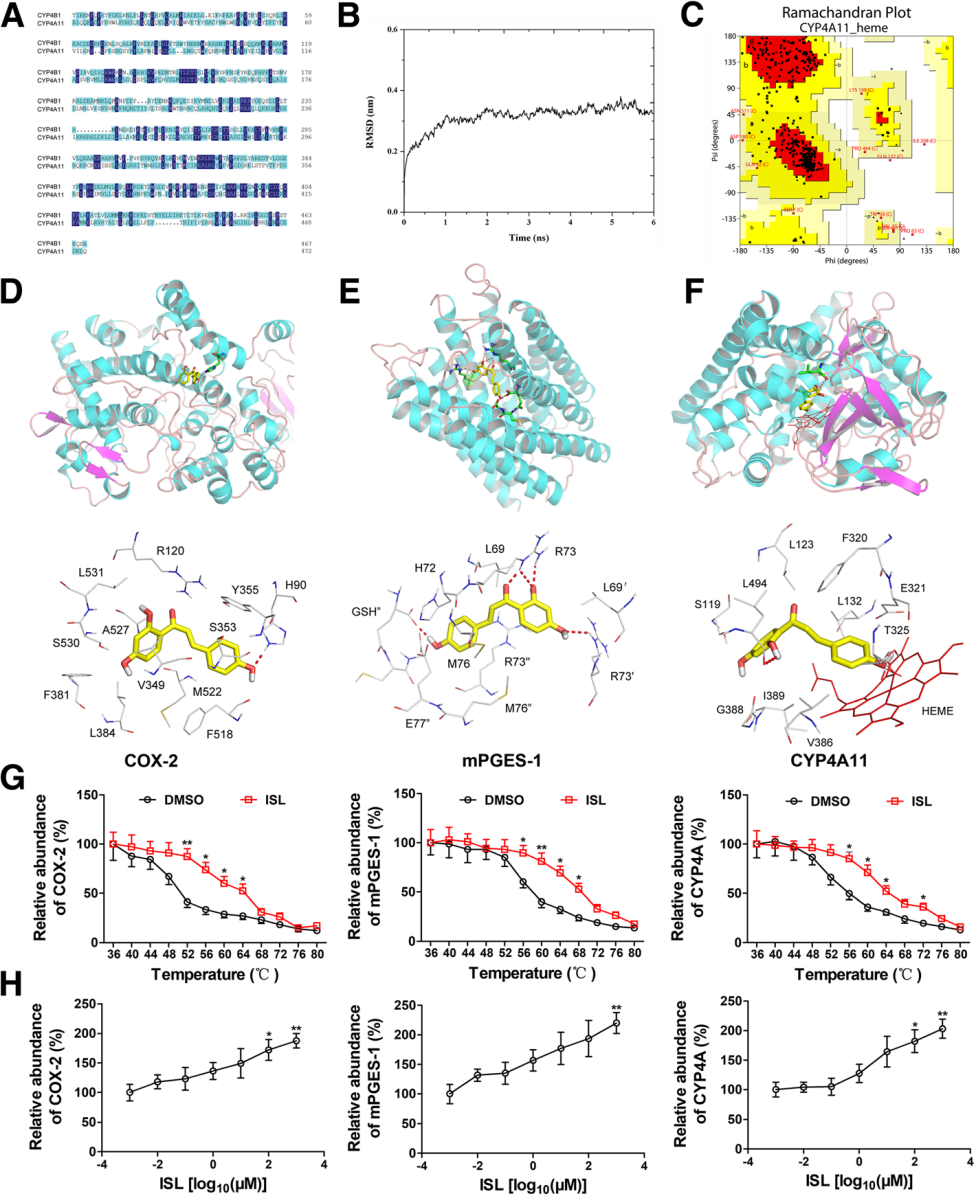
Structure-based virtual screening against COX-2, mPGES-1 and CYP4A11 3D models. **a** Sequence alignment results between CYP4A11 and template CYP4B1. The amino acid residues were colored by the Clustal method in Geneious. The dark blue color indicates conserved residues and light blue color indicates a semi-consereved substitution. **b** The backbone root mean square deviation (RMSD) values of the CYP4A11 during the dynamic simulation. **c** Ramachandran plot of the CYP4A11 model showing the distribution of residues in favored (red), allowed (yellow) and outlier (white) regions. **d** A structural view of the interaction of isoliquiritigenin (ISL) with COX-2. ISL is shown as a yellow stick representation. Details on the binding site interactions are shown in the down panel. Non-polar hydrogen atoms are hidden for clarity. Potential intermolecular hydrogen bonds are shown as red dashed lines. **e** The structural view of the interaction of ISL with mPGES-1. **f** The structural view of the interaction of ISL with CYP4A11. Heme is drawn as a red line representation. **g** ISL treatment (20 μM) increases the thermal stability of COX-2, mPGES-1 and CYP4A11 in cell lysates as measured by the temperature-dependent cellular thermal shift assay (*n* = 3). **h** ISL treatment increases the thermal stability of COX-2, mPGES-1 and CYP4A11 in cell lysates as measured by the concentration-dependent cellular thermal shift assay at 52 °C (*n* = 3)

Given that the crystal structure of CYP4A11 is not included in the Protein Data Bank, and CYP4A11 had the highest sequence identity (56%) with CYP4B1 (Fig. 1a), the CYP4B1 (PDB id: 5T6Q) was used as a template to build the 3D structure of CYP4A11. After 2 ns of simulation, the root means square deviation (RMSD) value of the CYP4A11 tended to be convergent with fluctuations around 3.5 Å (Fig. 1b). Furthermore, PROCHECK showed that 99.5% of the residues were located in the allowed regions (83.3% most favored) and only 0.5% (2 residues) outside the allowed regions (Fig. 1c). Verify 3D also showed that 85.3% of the residues had an averaged 3D-1D score > 0.2. These results indicate that the refined CYP4A11 3D model is reliable.

Next, by virtual screening against the crystal structure of human COX-2 (PDB id: 5IKT) and human mPGES-1 (PDB id: 3DWW), and the 3D structure of CYP4A11, we narrowed our interests from a total of 36,043 compounds (TCM Database) to 17 top flavonoids (Additional file 1: Table S1). Zebrafish screening was then undertaken to evaluate the antiangiogenic activity of the 17 flavonoids. The EC_50_ values, reported in Additional file 1: Table S1, showed that 11 out of the 17 flavonoids had moderate antiangiogenic activities, with EC_50_ values ranging from 5.9 to 38.1 μM. Among them, we observed the most promising compound ISL (ZINC03869608) with an EC_50_ value of 5.9 μM. Furthermore, ISL exhibited strong interaction with COX-2 (8.32), mPGES-1 (7.11) and CYP4A11 (7.61), respectively. The binding mode of ISL was predicted by the Surflex-Dock program. We found that ISL interacted with H90 of COX-2 through a hydrogen bond (H-bond) (Fig. 1d). Apart from H-bond interaction, ISL also formed several hydrophobic interactions with key catalytic site residues such as R120, V349, S353, Y355, F381, L384, F518, M522, A527, S530 and L531 of COX-2. ISL formed H-bond with R73, R73’ and GSH" of mPGES-1 and interacted with L69, H72, M76, L69’, R73”, M76” and E77” through a lipophilic interaction (Fig. 1e). We also found that ISL formed stable H-bond with L494 and hydrophobic interactions with S119, L123, L132, F320, E321, T325, V386, G388, I389 and L494 of CYP4A11 (Fig. 1f).

To further validate the binding of ISL to COX-2, mPEGS-1 and CYP4A11, we performed the CETSA in human U87 cells. The thermal stability of human COX-2, mPEGS-1 and CYP4A11 in the ISL-treated U87 cells was enhanced with the increased temperatures (36 to 80 °C) and ISL concentration (0.001 to 1000 μM) (Fig. 1g and h), suggesting a direct interaction between ISL and human COX-2, mPEGS-1 and CYP4A11. Next, we found that ISL inhibited COX-2, mPGES-1 and CYP4A11 activities with the IC_50_ values of 8.1 μM, 17.4 μM and 10.3 μM, respectively (Additional file 2: Figure S1A-C). We also examined the specificity of ISL for CYP4A11 and found that ISL was approximately 2.59- and 3.61-fold selective for CYP4A11 over CYP4B1 and its homolog CYP4V2, respectively (Additional file 2: Figure S1C-E). These data suggest that ISL is a potential inhibitor of mPGES-1, COX-2 and CYP4A11.

### 20-HETE or PGE_2_ reversed the antiangiogenic effects of the ISL in the zebrafish embryo and rabbit cornea

**Fig. 2:**
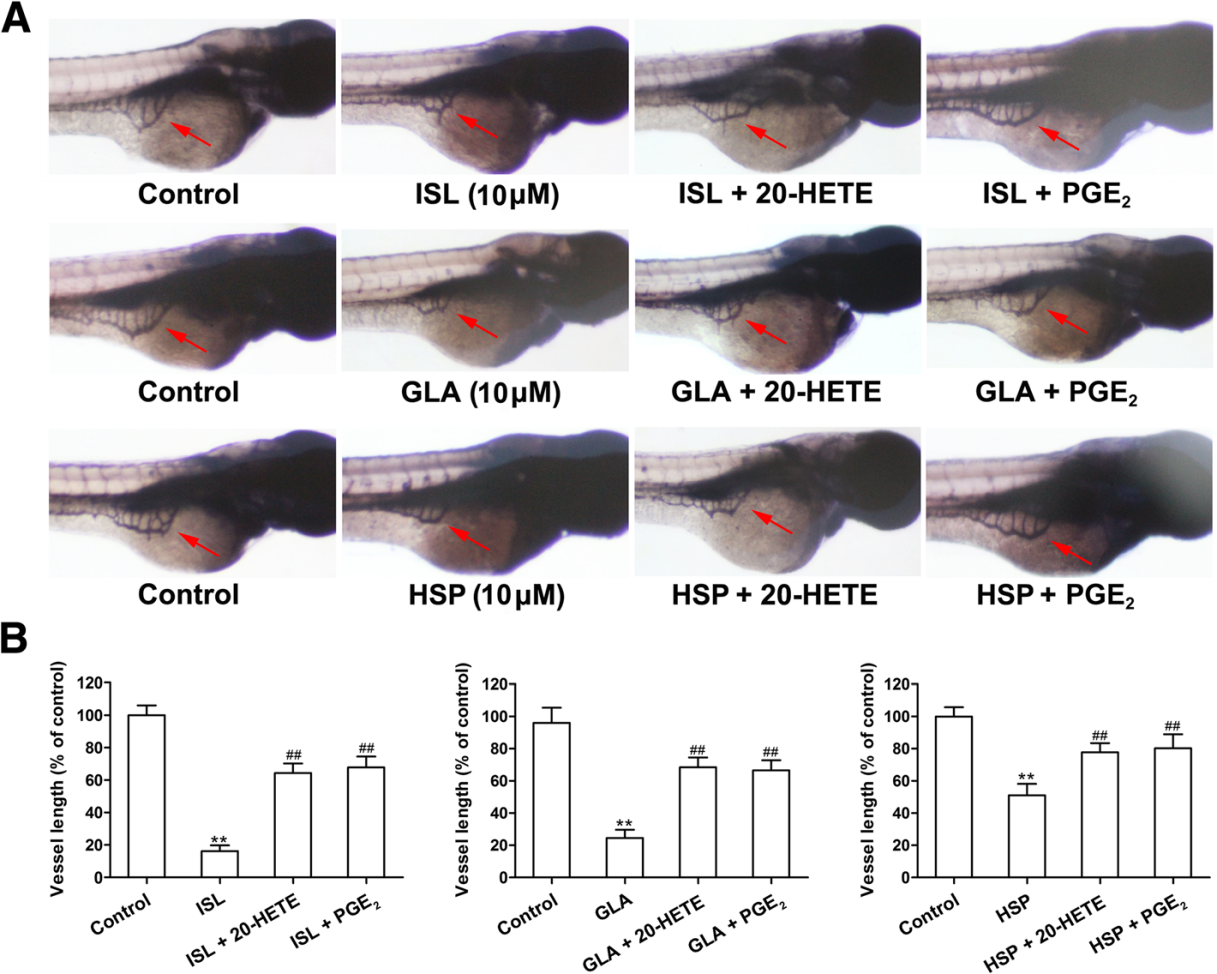
20-HETE or PGE_2_ partly reverses the antiangiogenic activities of the screened molecules in the zebrafish embryo. **a** The zebrafish embryos in 96-well microplate were treated with the indicated concentrations of compounds [isoliquiritigenin (ISL), glabridin (GLA) and hesperetin (HSP), 10 μM] alone, the compounds (10 μM) plus 20-HETE or PGE_2_, and vehicle for 48 h. Selected images of angiogenesis in the zebrafish embryo in different groups as shown in (**a**); Average vessel length for each group (**b**). The values are presented as the mean ± SEM, *n* = 20. ^*^P < 0.05; ^**^P < 0.01 vs. control; ^#^*P* < 0.05; ^##^*P* < 0.01 vs. ISL, GLA or HSP-treated groups

**Fig. 3:**
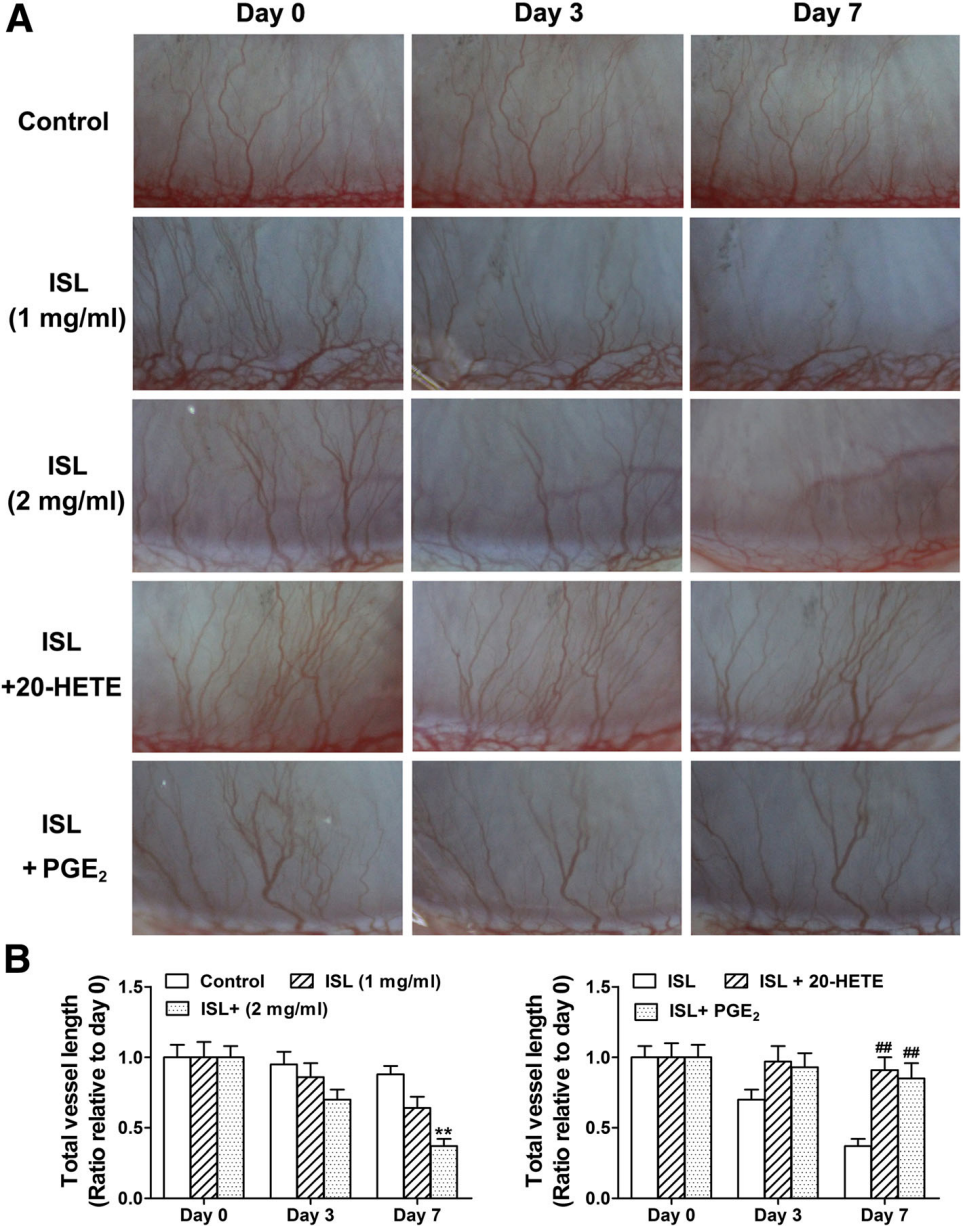
20-HETE or PGE_2_ partly reverses the antiangiogenic activities of isoliquiritigenin (ISL) in the rabbit cornea. A 7–0 black silk was used to pass the rabbit corneal stroma 1 mm from the horizontal to the limbus with a length of 3 mm to induce corneal neovascularization model. One week after suturing, the suture was removed, and the flavonoid ISL (1 and 2 mg/ml), ISL (2 mg/ml) plus 20-HETE or PGE_2_, and vehicle were performed twice a day for a week in eye drops. Photographs were taken with a stereomicroscope at day 0, 3 and 7 after treatment (**a**), and the vessel areas were quantified using image pro plus 6.0 (**b**). The values are presented as the mean ± SEM, *n* = 6. ^*^P < 0.05; ^**^P < 0.01 vs. control; ^#^*P* < 0.05; ^##^*P* < 0.01 vs. ISL or GLA-treated groups

We investigated whether the flavonoid ISL inhibits angiogenesis in the zebrafish embryo angiogenesis model through the downregulation of CYP4A and COX-2/mPGES-1 signaling, and found that exogenous addition of 20-HETE or PGE_2_ partly reversed the antiangiogenic activities of ISL (Fig. 2). Given that rabbit corneal neovascularization model was widely adopted for studying angiogenesis [24], we then measured the antiangiogenic activities of ISL in the model. As shown in Fig. 3, ISL reduced corneal neovascularization in a time (0, 3 and 7 days)-dependent manner when compared with the control. Conversely, 20-HETE or PGE_2_ partly reversed the antiangiogenic effects. These results suggest that the flavonoid ISL restrain the zebrafish embryo and rabbit corneal neovascularization, at least partly through the inhibition of CYP4A and COX-2/mPGES-1 signaling.

### Flavonoid ISL prolongs survival, delays growth and normalizes vasculature in the C6 glioma

**Fig. 4:**
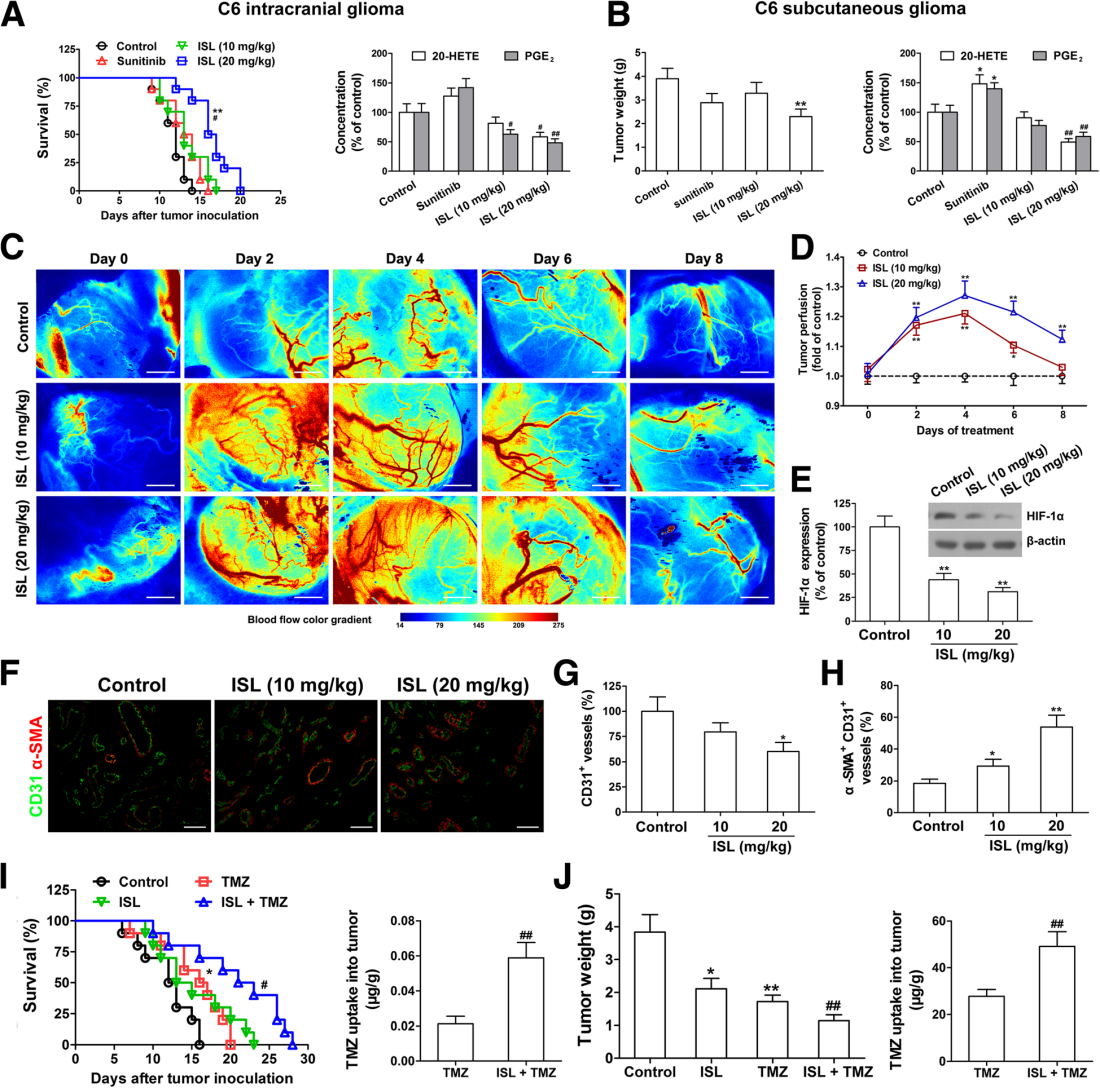
Isoliquiritigenin (ISL) prolongs survival, delays growth and induces vessel normalization in C6 gliomas with the decreased intratumoral level of 20-HETE and PGE_2_. **a** In the C6 intracranial glioma model, the survival time of the rats (*n* = 10) injected intraperitoneally with ISL (10 and 20 mg/kg), Sunitinib (80 mg/kg) or vehicle was measured, and 20-HETE and PGE_2_ in the tumor tissues from each group were determined at day 12 by LC-MS/MS or ELISA. **b** In the C6 subcutaneous glioma model, rat C6 glioma cells (5 × 10^6^) were injected subcutaneously into the right flank of Wistar rats. When tumors reached a size of about 100 mm^3^, the rats (*n* = 8) received ISL (10 and 20 mg/kg), Sunitinib (80 mg/kg) or vehicle by intraperitoneal injection once daily for a week. Tumor weight was measured, and 20-HETE and PGE_2_ were determined by LC-MS/MS or ELISA. The values are presented as the mean ± SEM, ^*^P < 0.05, ^**^P < 0.01 vs. control, ^**#**^*P* < 0.05, ^**##**^*P* < 0.01 vs. sunitinib (80 mg/kg)-treated group. **c**-**h** Rat C6 glioma cells (5 × 10^6^) were injected subcutaneously into the right flank of Wistar rats. When tumors reached a size of about 100 mm^3^, the rats (*n* = 8) received ISL (10 and 20 mg/kg) or vehicle by intraperitoneal injection once daily for a week. Tumor perfusion at day 0, 2, 4, 6 and 8 was measured using a laser Doppler analyzer. Scale bars, 2 mm (**c**). The quantitative analysis showed the relative level of tumor perfusion (**d**). After sacrificing the rats at day 8, hypoxia induced factor (HIF)-1α in the tumor tissues was measured by Western blot (**e**). Double staining for CD31 (green) and α-SMA (red) in the tumor tissues was shown. Scale bars, 50 μm (**f**-**h**). **i** In the C6 intracranial glioma model, the survival time of the rats (*n* = 10) injected intraperitoneally with ISL (20 mg/kg), temozolomide (TMZ, 20 mg/kg), ISL (20 mg/kg) plus TMZ (20 mg/kg) or vehicle was measured, and TMZ uptake into tumor tissues was determined at day 12 by high performance liquid chromatography (HPLC). **j** In another experiment, rat C6 glioma cells (5 × 10^6^) were injected subcutaneously into the right flank of Wistar rats. When tumors reached a size of about 100 mm^3^, the rats (*n* = 8) received ISL (20 mg/kg), TMZ (20 mg/kg), ISL (20 mg/kg) plus TMZ (20 mg/kg) or vehicle by intraperitoneal injection once daily for a week. Tumor weight was measured, and TMZ uptake into tumor tissues was determined by HPLC. The values are presented as the mean ± SEM, ^*^P < 0.05, ^**^P < 0.01 vs. control, ^**#**^*P* < 0.05, ^**##**^*P* < 0.01 vs. TMZ (20 mg/kg)-treated group

We first treated rats bearing intracranial C6 with liposomal ISL, sunitinib or blank liposome. As shown in Fig. 4a, ISL (20 mg/kg) significantly increased the survival (median 18.5 d) in C6-bearing rats compared with the sunitinib (13.5 d) or control-treated rats (11.5 d), accompanied by the decreased intratumoral level of 20-HETE and PGE_2_. Conversely, sunitinib (80 mg/kg) significantly increased the intratumoral level of 20-HETE and PGE_2_ compared with the control (Fig. 4a). Given the significant improvement in survival in rats treated with ISL over sunitinib, we examined the impact of treatment on growth in the subcutaneous C6 glioma. As shown in Fig. 4b, ISL significantly decreased tumor weight and the intratumoral level of 20-HETE and PGE_2_. In contrast, sunitinib increased the intratumoral level of 20-HETE and PGE_2_ without influencing tumor weight (Fig. 4b). These results suggest that ISL prolongs survival and delays growth in the C6 glioma.

Normalization of tumor vasculature prolongs survival in glioma [16]. To determine whether ISL prolongs survival in glioma through normalizing the tumor vasculature, we assessed time-course effects on tumor perfusion using laser Doppler analysis. In the subcutaneous C6 model, ISL (10 and 20 mg/kg) improved tumor perfusion at day 2 of treatment by 17.0 and 19.6%, respectively. A steady increase in tumor perfusion until day 4 by 21.0 and 27.1% in the ISL (10 and 20 mg/kg)-treated groups was observed, followed by a sharp decrease till day 8 by 20.4 and 17.3%, respectively. In contrast, tumor perfusion in the control group increased after 2 days by 7.5%, and increased higher by 9.5% at day 4, followed by a decrease by 6.5% till day 8 (Fig. 4c and d). We then determined tumor hypoxia by analysis for hypoxia-inducible factor (HIF)-1α. As expected, HIF-1α expression in C6 glioma was decreased in response to ISL treatment as compared with the control (Fig. 4e). Since pericyte coverage improves vessel maturation [27], we double stained for the endothelial cell marker CD31 and the pericyte marker α-smooth muscle actin (α-SMA), and observed the decreased microvessel density and the increased pericyte coverage of tumor vessels in the ISL (10 and 20 mg/kg)-treated groups when compared with the control (Fig. 4f-h).

TMZ is the standard chemotherapy agent for glioblastoma, so we evaluated whether ISL enhances the efficacy of TMZ therapy [28]. We found that the ISL (20 mg/kg) plus TMZ therapy significantly increased rat survival (median 22 d) compared with the TMZ (17 d) or control (12 d) in the intracranial C6 model (Fig. 4i). In addition, the dual therapy significantly increased TMZ concentration in the glioma tissues when compared with the TMZ alone (Fig. 4i). In the subcutaneous C6 model, dual therapy significantly inhibited tumor growth, whereas increased intratumoral level of TMZ compared with TMZ monotherapy (Fig. 4j). These data indicate that ISL induces vascular normalization and thereby enhances the efficacy of TMZ.

### Inhibition of COX-2, mPGES-1 and CYP4A by ISL decreases FGF-2, TGF-β and VEGF production via angiogenic-Akt signaling

**Fig. 5:**
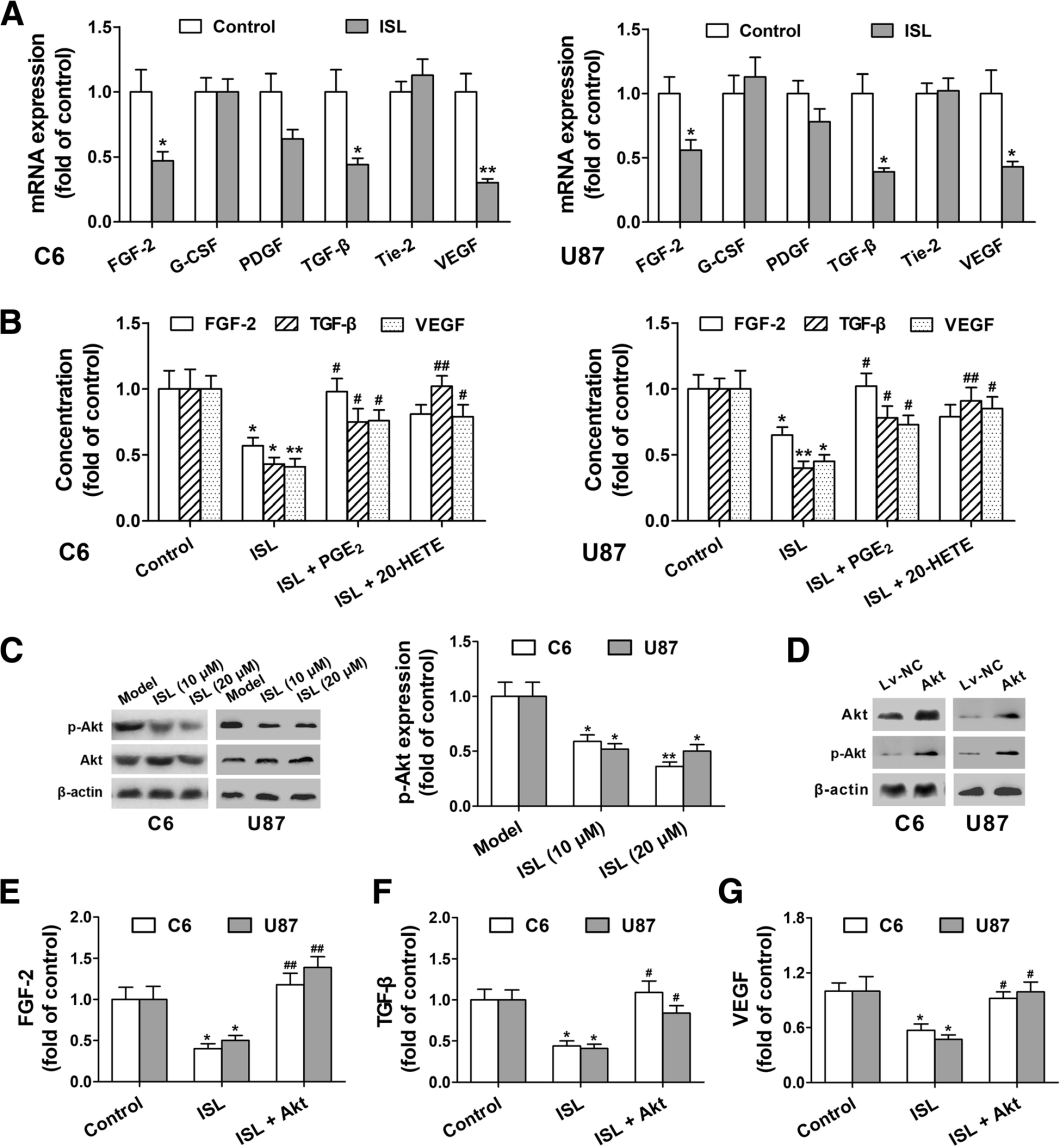
Inhibition of COX-2, mPGES-1 and CYP4A by ISL decreases FGF-2, TGF-β and VEGF production via Akt signaling. **a** FGF-2, G-CSF, PDGF, TGF-β, Tie-2 and VEGF in the C6 and U87 glioma cells were determined by qPCR. **b** FGF-2, TGF-β and VEGF in the C6 and U87 glioma cells were determined by ELISA. **c** Akt and p-Akt were determined in C6 and U87 glioma cells by Western blot. **d** The effects of Akt overexpression on Akt and p-Akt protein level in C6 and U87 glioma cells were measured by Western blot. **e**-**g** FGF-2, TGF-β and VEGF in the C6 and U87 glioma cells were determined by ELISA. Each value represents the mean ± SEM of three independent triplicate experiments. ^*^P < 0.05, ^**^P < 0.01 vs. control, ^**#**^*P* < 0.05, ^**##**^*P* < 0.01 vs. ISL (20 μM)-treated group

Glioma cells secrete a wide variety of cytokines that are associated with tumor angiogenesis. Thus, we used qPCR to investigate the effects of ISL on the glioma cell-derived angiogenic factors. Given that glioma cells in culture show negligible 20-HETE synthesis, CYP4A was overexpressed by transfection with CYP4A11 lentiviral activation particle in the C6 and U87 cells [29]. As shown in Fig. 5a, FGF-2, TGF-β and VEGF mRNA expression were decreased by ISL in the C6 and U87 cells, but G-CSF, platelet-derived growth factor (PDGF) and Tie-2 not. Conversely, exogenous addition of PGE_2_ or 20-HETE partly reversed these effects of ISL (Fig. 5b).

PI3K/Akt signaling is crucial for tumor angiogenesis [16]. We found that ISL decreased p-Akt without influencing Akt in the C6 and U87 glioma cells (Fig. 5c). To investigate whether ISL blocks FGF-2, TGF-β and VEGF production via Akt signaling, Akt was overexpressed by transfection with Akt lentiviral activation particle in the C6 and U87 glioma cells (Fig. 5d). We found that Akt overexpression reversed ISL-induced decrease in FGF-2, TGF-β and VEGF production (Fig. 5e and g). These results suggest that COX-2, mPGES-1 and CYP4A inhibition in glioma cells by ISL down-regulates FGF-2, TGF-β and VEGF production via Akt signaling.

### Flavonoid ISL inhibits Akt phosphorylation by ceRNA effect of miR-194-5p and lncRNA NEAT1

**Fig. 6:**
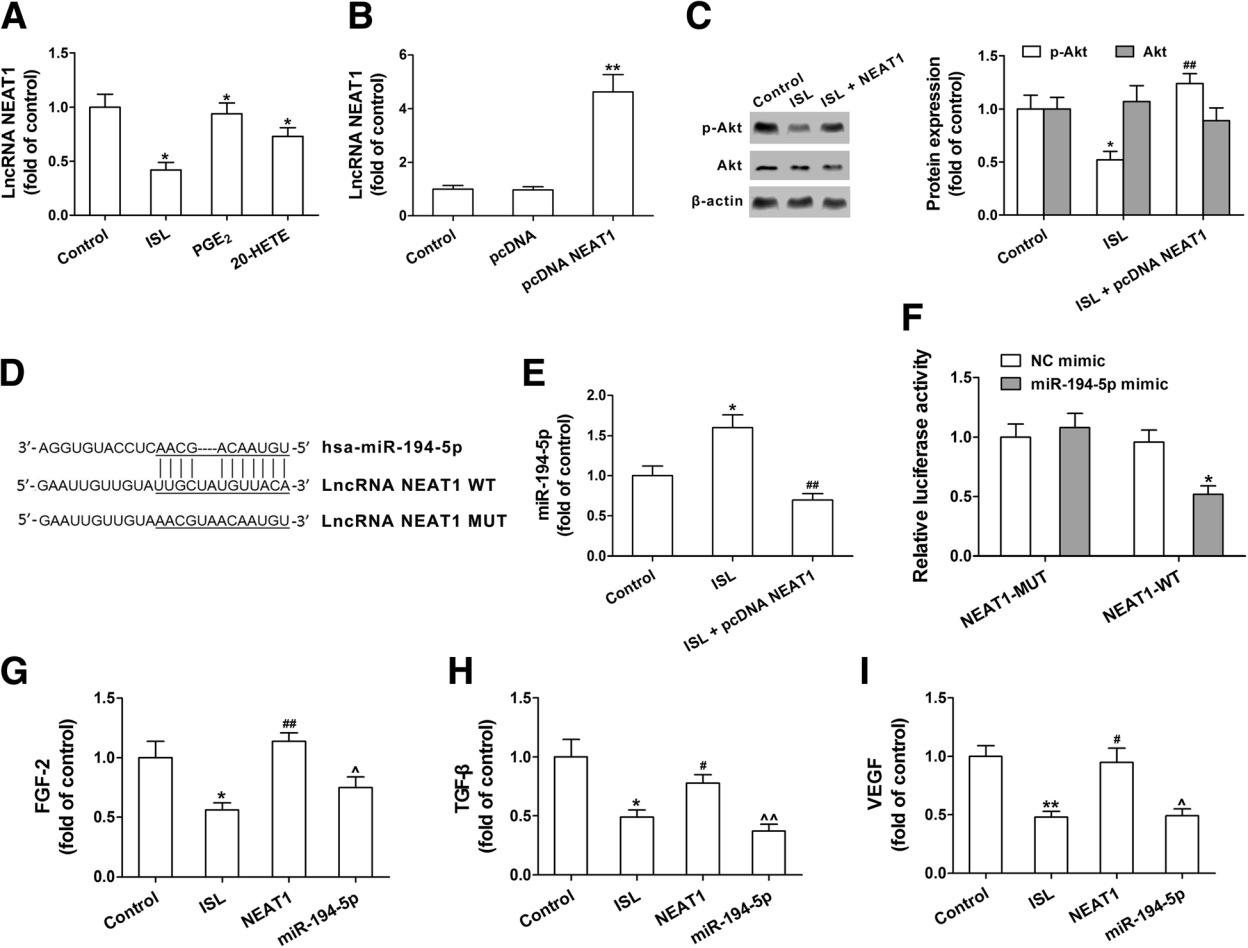
Isoliquiritigenin (ISL) inhibits Akt phosphorylation by ceRNA effect of miR-194-5p and lncRNA NEAT1. **a** The levels of lncRNA NEAT1 in the U87 glioma cells was measured by qPCR. **b** The U87 glioma cells were transfected with pcDNA-NEAT1 for lncRNA NEAT1 overexpression. LncRNA NEAT1 expression was measured using qPCR. **c** Akt and p-Akt expression was measured by Western blotting. **d** The putative miR-214-5p binding 3′ UTR of lncRNA NEAT1 (NEAT1-WT) and lncRNA NEAT1 mutation sequence (NEAT1-MUT). **e** The expression of miR-214-5p in the U87 glioma cells transfected with pcDNA-NEAT1 or negative control (pcDNA). **f** Luciferase reporter plasmid containing NEAT1-WT or NEAT1-MUT was transfected into the U87 glioma cells together with miR-214-5p mimic in parallel with NC. The luciferase assay was conducted. **g**-**i** FGF-2, TGF-β and VEGF in the U87 glioma cells were determined by ELISA. Each value represents the mean ± SEM of three independent triplicate experiments. ^*^P < 0.05, ^**^P < 0.01 vs. control, ^**#**^*P* < 0.05, ^**##**^*P* < 0.01 vs. ISL (20 μM)-treated group. ^^^ *P* < 0.05, ^^^^ *P* < 0.01 vs. AKT2-WT plus miR-194-5p mimic, NEAT1-WT plus miR-194-5p mimic or pcDNA NEAT1 plus miR-194-5p mimic; ^&^ *P* < 0.05, ^&&^ *P* < 0.01 vs. pcDNA

Growing evidence suggests that lncRNA NEAT1 are involved in the regulation of Akt phosphorylation in glioma [30]. We found that lncRNA NEAT1 was significantly decreased by ISL (20 μM) in the U87 glioma cells (Fig. 6a). Conversely, exogenous addition of PGE_2_ or 20-HETE partly reversed the effect of ISL (Fig. 6a). To investigate whether ISL blocks Akt phosphorylation via lncRNA NEAT1, lncRNA NEAT1 were overexpressed by transfection with pcDNA-NEAT1 in the U87 glioma cells (Fig. 6b). We found that lncRNA NEAT1 overexpression reversed ISL-induced decrease in p-Akt expression (Fig. 6c). Furthermore, through bioinformatics analysis by Starbase v2.0 program (http://starbase.sysu.edu.cn/), we found that miR-194-5p contains one conserved target site of lncRNA NEAT1 (Fig. 6d). Next, we determined the effects of lncRNA NEAT1 on miR-194-5p expression, and found that ISL upregulated the miR-194-5p expression in the U87 glioma cells. This effect was attenuated by lncRNA NEAT1 overexpression (Fig. 6e). Luciferase assay also showed that overexpression of miR-194-5p decreased the luciferase activity of lncRNA NEAT1 as compared with the NC mimic group. Conversely, transfection of mutated lncRNA NEAT1 together with miR-194-5p mimic did not significantly influence luciferase activity (Fig. 6f). The lncRNA NEAT1 overexpression significantly reversed the decreased FGF-2, TGF-β and VEGF production induced by ISL in the U87 glioma cells, but miR-194-5p mimic attenuated these effects (Fig. 6g-i). These results suggest that inhibition of COX-2, mPGES-1 and CYP4A by ISL blocks Akt phosphorylation and decreases FGF-2, TGF-β and VEGF production by ceRNA effect of miR-194-5p and lncRNA NEAT1.

## Discussion

Flavonoid ISL has already proved useful for the development of antiangiogenesis agents [14]. Our previous study showed that ISL decreased the gene expression of COX-2 and CYP 4A11 and production of PGE_2_ and 20-HETE in MDA-MB-231 and BT-549 human breast cancer cells [31]. In the present study, two novel observations have been made. First, we have provided direct evidence that ISL is a potent COX-2, mPGES-1 and CYP4A11 inhibitor, and thereby blocks angiogenesis and induces vascular normalization in glioma through downregulation of FGF-2, TGF-β and VEGF. To our knowledge, this is the first study that directly demonstrates the antiangiogenic activities of triple inhibition of COX-2, mPGES-1 and CYP4A11 by ISL. Second, we demonstrate that inhibition of COX-2, mPGES-1 and CYP4A by ISL blocks the angiogenic Akt signaling in glioma through ceRNA effect of miR-194-5p and long non-coding RNA NEAT1. Our finding may represent a potential therapeutic strategy for human glioma angiogenesis.

Arachidonic acid (AA) is liberated from the cellular membranes by cytoplasmicphospholipaseA2 (PLA2), and further metabolized through COX-2/mPGES-1 and CYP4A pathways to eicosanoids, including PGE_2_ and 20-HETE [11]. PGE_2_ and 20-HETE are abundantly produced by various tumors, and play crucial roles in tumor angiogenesis [6, 7]. HET0016, a 20-HETE synthesis inhibitor, was found to inhibit the angiogenic responses to EGF, VEGF and FGF-2 in rats [9]. COX-2 inhibition potentiates antiangiogenic cancer therapy in preclinical models [6]. However, inhibition of COX pathway would shunt AA metabolism to CYP4A pathways, thereby decreasing the efficacy and exacerbating adverse effects [32]. Inhibition of both COX pathway and CYP450 pathway elicits an additive therapeutic efficacy and improved safety profile compared with pure COX blockers [32, 33]. Herein, we demonstrated that the screened candidate flavonoid ISL is a potent COX-2, mPGES-1 and CYP4A11 inhibitor, with an IC_50_ of 33.7, 18.6 and 14.3 μM on COX-2, mPGES-1 and CYP4A11, respectively. Furthermore, inhibition of COX-2/mPGES-1 and CYP4A by ISL blocked glioma angiogenesis in a rat C6 glioma model. These data suggest that inhibition of COX-2, mPGES-1 and CYP4A-mediated AA metabolism blocks tumor angiogenesis, could be a novel strategy for glioma therapy.

Clinically, one of the most important treatment modalities for glioma is antiangiogenic drugs targeting VEGF and its receptor 2 (VEGFR2) [34]. Unfortunately, the survival benefits of antiangiogenic therapy have been limited due to intrinsic/acquired resistance [35]. Sunitinib, an oral pan-VEGF receptor tyrosine kinase inhibitor, fails to improve survival in a phase II trial in patients with rGBM [36]. FGF-2 and TGF-β are produced by tumor cells, and play important roles in antiangiogenic resistance [35, 37]. The combination of TGF-β receptor 1 antagonist LY2157299 and VEGF antibody B20 exhibits additive antiangiogenic and antitumor growth effects [35]. Herein, we demonstrated that inhibition of COX-2, mPGES-1 and CYP4A by ISL decreased FGF-2, TGF-β and VEGF production, and blocked glioma angiogenesis. These data suggest that downregulation of COX-2/mPGES-1 and CYP4A by ISL could provide effective and safe way against tumor angiogenesis.

The PI3K/Akt pathway has been shown to regulate the production of angiogenic cytokines in tumor cells [9]. The functions of lncRNAs and miRNAs in tumor angiogenesis have drawn more and more attention [38, 39]. The miR-194-5p in glioma cells play an essential role in proliferation and migration [40]. LncRNA NEAT1 is notably increased in glioma [41], and promotes the prostate cancer cell growth through the Akt pathway [30]. Herein, we demonstrated that downregulation of lncRNA NEAT1 by ISL inhibited Akt phosphorylation by targeting miR-194-5p in the U87 glioma cells. In vitro inhibition of COX-2, mPGES-1 and CYP4A by ISL decreased FGF-2, TGF-β and VEGF production in the C6 and U87 glioma cells, accompanied with the downregulation of p-Akt expression. These effects were reversed by overexpression of Akt. These data suggest that reprogramming COX-2, mPGES-1 and CYP4A mediated-AA metabolism in glioma by flavonoid ISL inhibits the angiogenic Akt- FGF-2/TGF-β/VEGF signaling in glioma through ceRNA effect of miR-194-5p and lncRNA NEAT1.

Biological screening alone is not likely to improve the productivity and speed of drug discovery, thus, combining in silico and biological screening is expected to have an increasing importance [42]. Flavonoids are prospective compounds for anti-cancer therapy because of their safety, cost-effectiveness, and feasibility of oral administration [43]. Accumulating evidence indicates that flavonoids can act as an inhibitor of tumor angiogenesis [44]. Flavonoid ISL inhibits adenoid cystic carcinoma and breast cancer angiogenesis [14, 45]. In this study, the 17 top flavonoids were selected from 36,043 compounds (TCM Database) through a “sequential” virtual screening against the COX-2, mPGES-1 and CYP4A11 3D model. The 17 candidates exhibited moderate antiangiogenic activities in a zebrafish model. Eleven flavonoids out of the 17 candidates (about 64.7%) showed stronger antiangiogenic activities, with EC_50_ values ranging from 5.9 to 38.1 μM. The most promising candidate ISL showed significantly antiangiogenic activity in the zebrafish and rabbit corneal models, which consistent with the results reported by Han et al. [46] and Jhanji et al. [47]. Furthermore, ISL normalized glioma vasculature and improved the efficacy of temozolomide therapy through COX-2/mPGES-1 and CYP4A-mediated VEGF signaling in the rat C6 glioma model. Taken together, these data reveal that the combination of in silico and zebrafish screening is an efficient strategy for screening multiple-target inhibitors from flavonoids to block angiogenesis.

## Conclusions

**Fig. 7:**
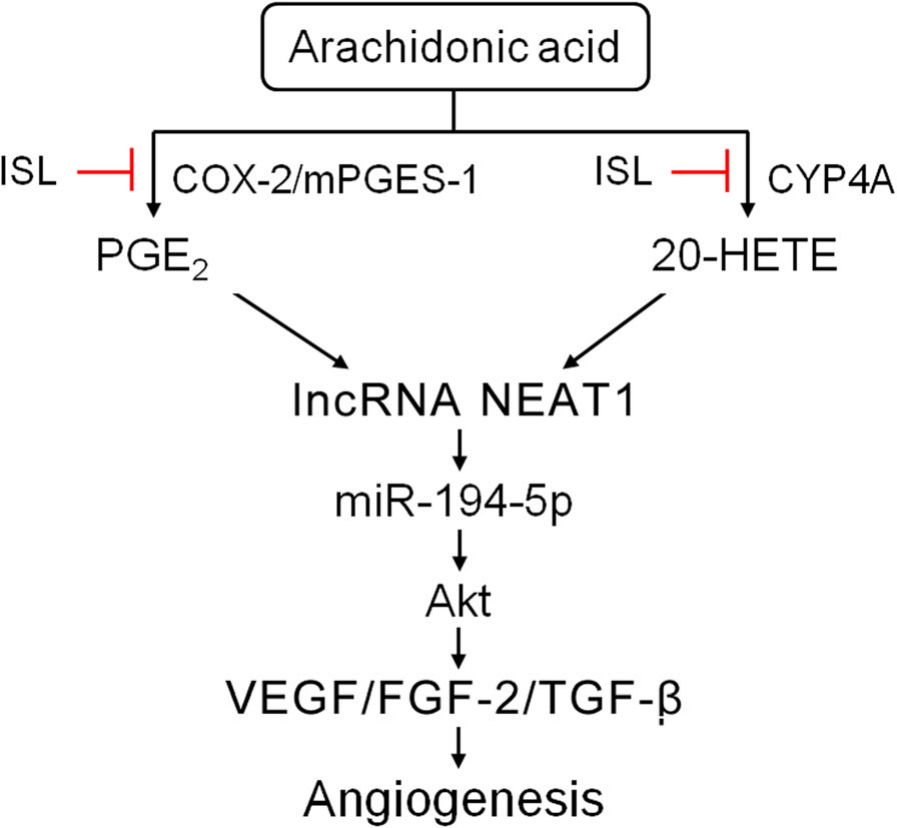
Scheme depicts the proposed mechanisms underlying the flavonoid isoliquiritigenin blocks the angiogenic Akt signaling through reprogramming COX-2, mPGES-1 and CYP4A mediated-AA metabolism in glioma by competing endogenous RNA effect of miR-194-5p and lncRNA NEAT1

Altogether, reprogramming COX-2, mPGES-1 and CYP4A mediated-AA metabolism in glioma by flavonoid ISL inhibits the angiogenic Akt- FGF-2/TGF-β/VEGF signaling through ceRNA effect of miR-194-5p and lncRNA NEAT1 (Fig. 7). Importantly, our study identifies a previously unknown ceRNA effect of miR-194-5p and lncRNA NEAT1 between the crosstalk of arachidonic acid (AA) metabolism with angiogenic Akt signaling in glioma. Our findings suggest that targeting inhibition of COX-2, mPGES-1 and CYP4A by ISL blocks glioma angiogenesis, and may serve as a novel strategy against glioma. These results also lay a solid foundation for the development of novel dietary flavonoids for the treatment of glioma in the near future.

## Additional files

Additional file 1: **Table S1.** The antiangiogenic effects of the top 17 flavonoids in zebrafish embryos. (DOCX 145 kb)

Additional file 2: **Figure S1.** Effect of isoliquiritigenin on COX-2, mPGES-1, CYP4A11, CYP4B1 and CYP4V2 enzymes.

## Data Availability

Data sharing not applicable to this article as no datasets were generated or analysed during the current study.

## Abbreviations

20-HETE: 20-hydroxyeicosatetraenoic acid
CYP: Cytochrome P450
HET0016: N-hydroxy-N-(4-butyl-2 methylphenyl)-formamidine
HIF-1α: Hypoxia-inducible factor-1α
LC-MS/MS: Liquid chromatographic-tandem mass spectrometry
MD: Molecular dynamics
MVD: Microvessel density
PDB: Protein data bank
RMSD: Root mean square deviation
VEGF: Vascular endothelial growth factor
α-SMA: α-smooth muscle actin
