# Supplementary material for: Inhibition of COX-2, mPGES-1 and CYP4A by isoliquiritigenin blocks the angiogenic Akt signaling in glioma through ceRNA effect of miR-194-5p and lncRNA NEAT1
Source: J Exp Clin Cancer Res. 2019 Aug 22;38:371. doi: 10.1186/s13046-019-1361-2 (PMC6704644; doi:10.1186/s13046-019-1361-2)
Supplement: Supplementary file 1 — Table S1. The antiangiogenic effects of the top 17 flavonoids in zebrafish embryos. (DOCX 145 kb) [file 13046_2019_1361_MOESM1_ESM.docx]

**Table. S1 The antiangiogenic effects of the top 17 flavonoids in zebrafish embryos.**

| **#** | ZINC ID | Structure | Total-Score | | | | EC_50_ (μM) |
| --- | --- | --- | --- | --- | --- | --- | --- |
|  |  |  | COX-2 | mPGES-1 | CYP4A11 | AVG |  |
| 1 | 03869608 |  | 8.3156 | 7.1128 | 7.6133 | 7.6806 | 5.9 |
| 2 | 05854640 |  | 7.3340 | 7.0128 | 8.1048 | 7.4839 | 9.1 |
| 3 | 95909868 |  | 8.3344 | 7.5770 | 7.2261 | 7.7125 | 12.6 |
| 4 | 05158935 |  | 6.8799 | 8.3952 | 7.0345 | 7.4365 | 13.6 |
| 5 | 13108861 |  | 10.1291 | 8.3797 | 8.6052 | 9.0380 | 15.8 |
| 6 | 14505561 |  | 8.1137 | 7.6212 | 8.1846 | 7.9732 | 19.8 |
| 7 | 14762530 |  | 7.8742 | 9.4066 | 7.9953 | 8.4254 | 21.2 |
| 8 | 85633024 |  | 6.2285 | 6.9016 | 9.8187 | 7.6496 | 21.6 |
| 9 | 14762534 |  | 8.9856 | 9.1045 | 6.9614 | 8.3505 | 29.4 |
| 10 | 00898788 |  | 9.2808 | 6.8910 | 6.2462 | 7.4727 | 34.6 |
| 11 | 95919428 |  | 9.5398 | 7.3427 | 6.7601 | 7.8809 | 38.1 |
| 12 | 13341174 |  | 8.1761 | 7.3656 | 8.0254 | 7.8557 | > 40.0 |
| 13 | 13341109 |  | 7.7366 | 6.6613 | 8.7652 | 7.7210 | > 40.0 |
| 14 | 85632999 |  | 8.7205 | 7.4863 | 6.8624 | 7.6897 | > 40.0 |
| 15 | 15115166 |  | 6.2193 | 7.9768 | 8.6224 | 7.6062 | > 40.0 |
| 16 | 95909876 |  | 7.5639 | 7.4899 | 7.5490 | 7.5343 | > 40.0 |
| 17 | 14780848 |  | 8.0209 | 7.2075 | 7.1487 | 7.4590 | > 40.0 |
