# Supplementary material for: Inhibition of COX-2, mPGES-1 and CYP4A by isoliquiritigenin blocks the angiogenic Akt signaling in glioma through ceRNA effect of miR-194-5p and lncRNA NEAT1
Source: J Exp Clin Cancer Res. 2019 Aug 22;38:371. doi: 10.1186/s13046-019-1361-2 (PMC6704644; doi:10.1186/s13046-019-1361-2)
Supplement: Supplementary file 2 — Figure S1. Effect of isoliquiritigenin on COX-2, mPGES-1, CYP4A11, CYP4B1 and CYP4V2 enzymes. [file 13046_2019_1361_MOESM2_ESM.docx]

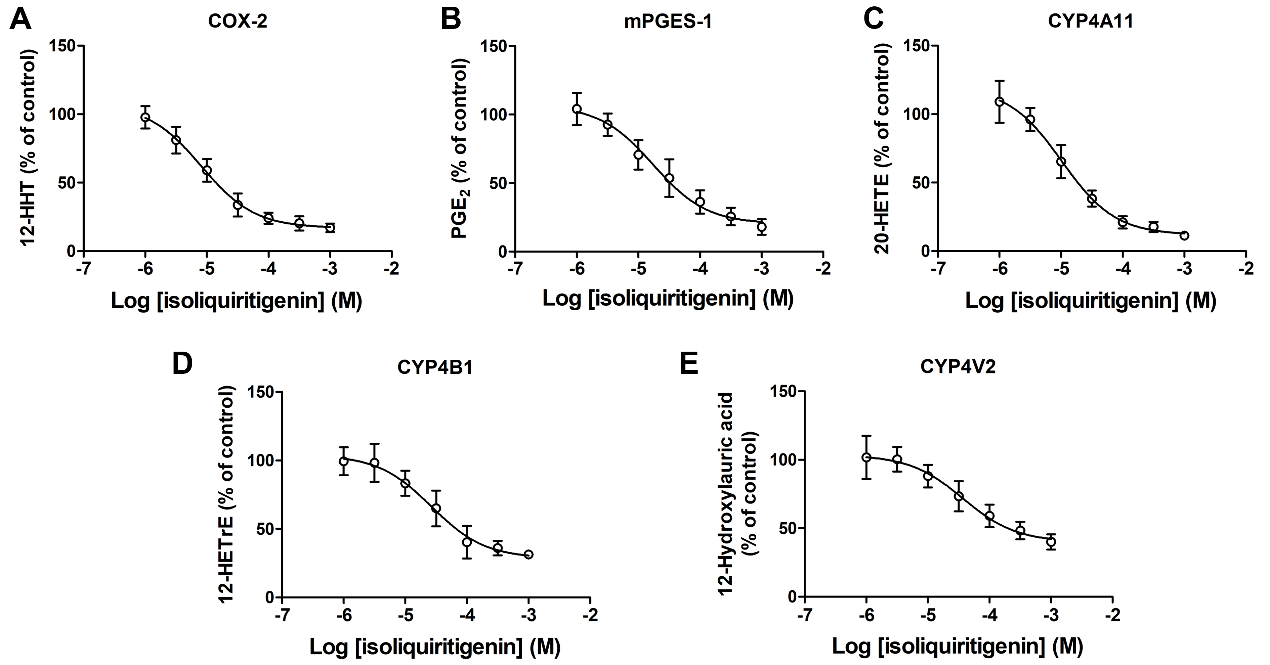


Figure S1. (A) Effect of isoliquiritigenin (1-1000 nM) on COX-2-catalyzed arachidonic acid was assayed. (B) Effect of isoliquiritigenin (1-1000 nM) on mPGES-1-catalyzed PGH_2_ was assayed. (C) Effect of isoliquiritigenin (1-1000 nM) on CYP4A11-catalyzed arachidonic acid was assayed. (D) Effect of isoliquiritigenin (1-1000 nM) on CYP4B1-catalyzed arachidonic acid was assayed. (E) Effect of isoliquiritigenin (1-1000 nM) on CYP4V2-catalyzed lauric acid was assayed. Each value represents the mean ± SEM of three independent triplicate experiments. 12-HHT, 12-Hydroxyheptadecatrenoic acid; 12-HETrE, 12-hydroxyeicosatrienoic acid.
